# Mobilizing the psychology evidence base for the treatment of pediatric chronic pain: The development, implementation, and impact of the Comfort Ability Program

**DOI:** 10.1002/pne2.12019

**Published:** 2020-06-16

**Authors:** Rachael Coakley, Simona Bujoreanu

**Affiliations:** ^1^ Division of Pain Medicine Department of Anesthesiology, Perioperative and Pain Medicine Boston Children’s Hospital Boston Massachusetts; ^2^ Department of Psychiatry Harvard Medical School Boston Children’s Hospital Boston Massachusetts

**Keywords:** child, chronic pain, evidence‐based, knowledge mobilization, parent, pediatric, psychological intervention

## Abstract

Over the past 20 years, our knowledge regarding evidence‐based psychological interventions for pediatric chronic pain has dramatically increased. Unfortunately, access to evidence‐based pain management interventions remains a challenge for many children and adolescents who suffer with persistent pain. Reducing patient burden and system‐level barriers to care are a central target of clinical innovations in pain treatment intervention. Psychological interventions are also increasingly focused on reducing biomedical biases that may inhibit attainment of services. While there are many new psychological interventions across an array of delivery platforms, few interventions have been systematically disseminated. This paper will highlight the translational research procedures that have informed the development and dissemination of the Comfort Ability Program (CAP), an interactive group‐based intervention teaching adolescents and their parents evidence‐based strategies to manage chronic or persistent pain. Now in its fifth year of dissemination, CAP has a demonstrated record of success with cross‐institutional implementation and sustainability at 18 hospitals across three countries. This paper reviews six dynamic and iterative phases of development, based on the Graham et al knowledge‐to‐action cycle (2006), that have guided the implementation and dissemination research for this program. The phases of CAP development include the following: (a) identifying knowledge and clinical gaps in care, (b) generating knowledge assets and implementation procedures, (c) evaluating clinical outcomes and system‐level processes, (d) developing and testing dissemination procedures, (e) expanding partnerships and monitoring knowledge use, and (f) sustaining knowledge use and continued innovation. This paper targets primarily health professionals and administrators and secondarily caregivers and the public at large.

## KNOWLEDGE DISSEMINATION

Across pediatric care, there remain significant gaps between scientific knowledge and routine clinical practice. In pediatric healthcare research, where funding resources are scarce, it is essential to build a conduit through which evidence‐based knowledge can actively inform clinical practice. Currently, it is estimated to take 17 years—an entire childhood—for evidence‐based interventions to trickle into practice.^1^, ^2^ There are several issues that contribute to this problem. From a research standpoint, the slow and arduous process of moving knowledge into practice via scientific publications and continuing education tools alone is insufficient to bring about systematic improvements in care.^1^ From a clinical practice perspective, overburdened practitioners often lack the time, system‐level support,  and specific expertise to generate and implement new interventions that address critical gaps in care. Finally, at the institutional level, bureaucratic organizations and complex healthcare systems may lack the flexibility or initiative to spearhead practice innovation. Moreover, institutions may be hindered  by implementation difficulties, relying on outmoded procedures for systematically implementing change.

The many challenges that thwart the knowledge‐to‐practice pipeline are, at a fundamental level, related to the lack of connectivity between two distinct populations: the evidence producers (scientists and researchers) and the evidence consumers (practitioners and healthcare institutions).^2^ While a formidable challenge, this identified chasm simultaneously presents opportunities for innovative translational and implementation science designed with intent to address both the clinical gaps in care and the system‐level demands that impede change. Pediatric psychologists who commonly straddle the two worlds of research and direct patient care may be particularly well‐poised to develop, evaluate, and disseminate interventions that can effectively help to close this gap.^3^

Closing the knowledge‐to‐practice gap in pediatric psychology means that clinical research must be tied to translational science. When there is a shared focus on dissemination and implementation procedures, knowledge creation is enhanced. This systematic approach boosts knowledge dissemination by employing research‐supported practices for sharing information with end‐users *and* the evaluation of the process by which this information is integrated into care. One theoretical model that fully incorporates the rigors of the translational science process with the mobilization of knowledge is the “knowledge‐to‐action cycle” by Graham et al. Within Graham’s model, knowledge creation occurs in a synergistic and evolving cycle that includes identification of the problem, adapting knowledge at the local context, assessing barriers to knowledge use, tailoring interventions and implementation procedures, monitoring knowledge use, evaluating outcomes, and sustaining knowledge use. Within this model, each phase of the knowledge creation process informs existing and future assets of the intervention. Moreover, the knowledge‐to‐action cycle describes how intervention effectiveness and implementation procedures are truly inter‐related processes. In a rapidly expanding field such as pediatric psychology, the goal of intentionally designing interventions for successive modification based on new research, on patient and practitioner response, and on system‐level needs is particularly compelling.

**Figure 1:**
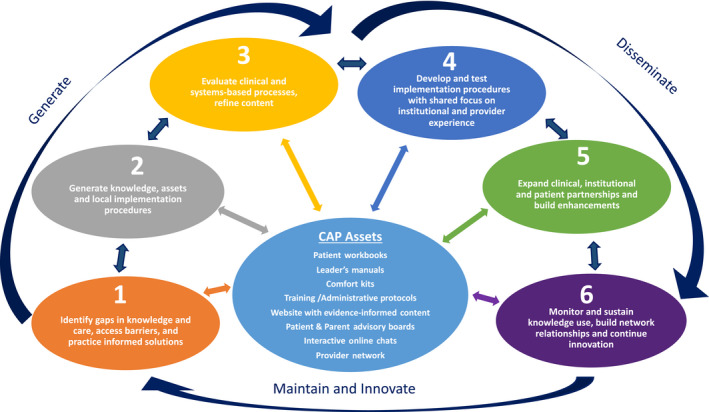
Comfort Ability Program (CAP) development process

The Comfort Ability Program (CAP) is a psychological intervention for adolescents with chronic pain and their parents, designed expressly to addresses several identified knowledge‐to‐practice gaps in the field of pediatric pain. CAP was developed to mobilize the psychology research evidence base for pediatric chronic pain, providing enhanced access to essential skills and strategies for patients and their parents. The main objective was to generate a targeted, patient‐centered, engaging intervention that could be systematically replicated to optimize knowledge mobilization. The phases of development for this program, including both the clinical and translational science components, are illustrated in Figure 1. While CAP is closely aligned with Graham’s theoretical knowledge‐to‐action cycle, the six phases in Figure 1 illustrate the real‐world translational science practice that has unfolded as CAP progressed through knowledge creation, evaluation, and dissemination. At the center of the figure are CAP’s assets, including the evidence‐supported clinical content (patient workbooks, leader manuals), training protocols, program enhancements (website, online chats), and partners (CAP network sites and patient‐partners). The outside spokes in this figure enumerate the phases of development of CAP over the last eight years. Notably, this figure highlights the hallmark feature of Graham’s theoretical model, the cyclical and bidirectional flow of information that influences knowledge creation and dissemination throughout each phase of development. By adhering to this dynamic process, CAP assets, implementation procedures, and dissemination protocols can continue to be tailored for optimal impact.

Importantly, while this article outlines a step‐wise translational science paradigm that has shaped CAP’s development, *it also highlights the iterative and evolving process of translational science more broadly*. The key phases of development that have informed the creation of CAP are described below. Each phase includes core knowledge mobilization strategies and goals, while integrating CAP’s real‐world experience with program development, research, and dissemination processes.

## 1 IDENTIFYING GAPS IN KNOWLEDGE AND CARE, ACCESS BARRIERS, AND PRACTICE‐INFORMED SOLUTIONS

The Comfort Ability Program was first developed in 2011 in response to a synthesis of the gap between scientific knowledge and practice in pediatric pain psychology. Specifically, there was a clear need for a psychological intervention that could provide accessible, non‐stigmatizing, supportive, psychoeducation, and hand‐on skills training. The program was further spurred by an urgent need to more efficiently provide psychological services to patients within the Pain Treatment Service at Boston Children’s Hospital where long waitlists for psychological services were a significant impediment to care. Recognizing that the clinical and system‐level challenges faced by one institution are often emblematic of more widespread difficulties, it was hoped that a well‐designed intervention could be scaled for implementation in a larger ecosystem of care. As will be discussed below, the original framework for Comfort Ability Program was informed by systematically identifying and evaluating gaps in knowledge and care, thoughtful consideration of access barriers,  and by taking an evidence‐informed approach to the development of the delivery mechanism to maximize impact.

### 1.1 Gaps in knowledge and care

Chronic and persistent pain in pediatrics is an increasingly common problem, with one in four adolescents experiencing at least one 3‐month episode of pain at some point in their development.^4^, ^5^ Persistent pain is known to be associated with functional impairment for pediatric patients (eg, school absenteeism, poor quality of life, development of internalizing disorders, increased severity of pain), and with psychosocial and psychological impact on the child’s family.^1^, ^6^, ^7^ Youth with chronic pain are high consumers of medical services,^8^ and when chronic pain is left untreated, there is increased risk for chronic pain in adulthood.^1^ Given the well‐established opioid epidemic for adults,^9^, ^10^, ^11^ the risk associated with untreated chronic pain in adolescents is especially concerning. Notably, the financial impact of managing chronic pain has been overwhelming for the healthcare system in the United States, costing an estimated 19.5 billion dollars annually, and ranking among the top three most expensive pediatric healthcare problems in the world.^8^, ^12^, ^13^, ^14^

Given all these factors, researchers and clinicians have made a widespread call to action for the implementation of evidence‐informed, psychologically based pain management interventions as part of a multidisciplinary approach to care.^1^, ^15^ Indeed, psychological interventions are well known to reduce pain and improve both physical and psychological functioning for pediatric patients.^16^, ^17^, ^18^, ^19^ Specifically, interventions such as those based on behavioral therapy, cognitive behavioral therapy (CBT), acceptance and commitment therapy (ACT), mindfulness‐based stress reduction, and self‐regulatory psychophysiological approaches such as biofeedback‐assisted relaxation, are well‐established and have been shown to be efficacious at reducing both pain intensity and disability and at improving psychological functioning.^16^, ^17^, ^18^, ^19^ Unfortunately, access to these types of psychological intervention remains challenging for many patients. Although a multidisciplinary treatment plan often includes recommendations for psychological services, research suggests that patients with pain much more commonly access physical therapy and medical intervention (ie, medications, additional testing) in multidisciplinary models of care.^20^

### 1.2 Access barriers

Working directly with a psychology provider who has expertise in pediatric pain psychology in a traditional one‐on‐one clinical practice can be an effective way for children with chronic pain to gain targeted intervention. Unfortunately, a primary access barrier is that there is a scarcity of pediatric behavioral health providers who have expertise in working with youth with chronic pain. Even when providers are available, there may be patient‐centered barriers such as scheduling conflicts, geographic limitations, and insurance or cost factors to consider.^14^, ^15^, ^20^

Another particularly challenging access barrier relates to biomedical biases and patient and parent exposure to psychological intervention. Research demonstrates that a positive past engagement with psychology is highly predictive of future engagement.^21^, ^22^ In one study assessing engagement in psychological intervention for pain, parents’ familiarity with psychological interventions (ie, biofeedback, hypnosis) was positively associated with their child’s engagement in treatment and predictive of positive expectation for treatment effectiveness.^20^ In other words, patients with pain are more likely to gain access to treatment if they and/or their parents have had exposure to psychological treatment in the past and have working knowledge about specific treatment modalities. Innovation in this field must address these fundamental access barriers within the knowledge‐to‐action process. Thus, an initial goal in developing CAP was to create (a) an accessible delivery model and (b) an opportunity for families to gain a positive exposure to psychological interventions for pain with the goal of enhancing future engagement with psychology if this was needed in the course of a child’s care.

### 1.3 Practice‐informed solutions

In the development of CAP, practice‐informed solutions were generated by asking the question, “How do we maximally share our evidence‐base while minimizing the identified access barriers that restrict engagement?” This question guided the framework for the program, helping to establish a unique format to enhance accessibility and support.

A well‐established way of making CBT and other empirically based interventions accessible to children and families, but still offering the personalization of working with a psychologist, as well as gaining support from peers, is using a brief group intervention. Within the pediatric literature, brief (<6 hours) psychoeducational and CBT interventions have demonstrated promising gains on variables such as self‐efficacy, self‐management, pain catastrophizing, family functioning, psychosocial well‐being, pain severity, school attendance, and feelings of hopefulness.^23^, ^24^, ^25^ Additionally, as parent training unequivocally enhances child outcomes in pediatric pain,^18^ one of CAP’s key practice‐informed goals was to design an intervention that simultaneously engaged parents and adolescents.

Condensing psychological interventions into a 1‐day workshop format has the added benefit of reducing scheduling barriers, increasing the likelihood of attracting families from a greater geographic area, and reducing the likelihood that patients will receive only a partial “dose” of the intervention, as this can occur when patients miss one or more group sessions in a multi‐week treatment approach.^26^ Moreover, evidence suggests that initial therapeutic‐related gains at 1‐month follow‐up could be maintained for as long as 1‐year after an intensive day‐long intervention.^25^, ^27^, ^28^ Importantly, brief psychoeducational interventions have a high rate of satisfaction from participants and are known to have similar benefits to patients with pain as compared with other structured CBT interventions ^29^, ^30^, ^31^.

To further reduce patient burden (ie, minimize loss of school and work time), increase participation, and minimize negative biases about psychology, research also suggests that CBT and psychoeducational interventions have been most effective when they are run on the weekends, are held outside of a mental health setting, and when they use non‐diagnostic titles.^32^, ^33^ The synthesis of this important research led to the generation of the non‐diagnostic name (The Comfort Ability) and the impetus to run the program on the weekend.

Comfort Ability Program was designed to address the needs of adolescents and families at various levels of readiness for change. It was initially designed as an entry‐level intervention, offering widely applicable psychoeducation, neuroscience education, and skills training. In clinical practice, it can function as a stand‐alone program, a roadmap for adolescents who are working with a mental health provider, or as a precursor for a more intense psychological treatment such as a day treatment program. Ongoing CAP research efforts are addressing the delivery of CAP across all such clinical situations and as intimate part of the knowledge synthesis and knowledge tool refinement.

## 2 GENERATE KNOWLEDGE, ASSETS, AND LOCAL IMPLEMENTATION PROCEDURES

**Figure 2:**
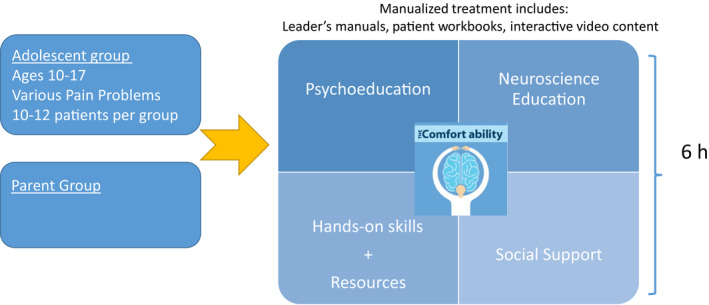
Comfort Ability Program (CAP) framework

The Comfort Ability Program was developed to serve a large number of families presenting for care at the local level. This included families who had an adolescent (ages 10‐17 years old) with a wide range of common types of persistent pain, such as headache, abdominal, neuropathic, and/or musculoskeletal pain, disease‐related pain, postinjury pain, or other kinds of persistent pain. CAP was designed to serve as first‐line intervention, or primary prevention for chronic pain, through targeted psychoeducation, neuroscience pain education, an array of cognitive behavioral therapy and other evidence‐based intervention skills, parent training skills, and additional science‐backed resources. Moreover, while CAP was nested within the Pain Treatment Service, it was intended to serve the whole hospital community. The goal was to enhance access to care more broadly. As such, CAP accepted direct referrals to the intervention from specialists such as neurologists, gastroenterologists, rheumatologists, and other specialty services where adolescents with persistent pain are likely to be treated. Figure 2 illustrates the general framework of CAP.

### 2.1 Generating knowledge and assets

While based primarily in CBT, the theoretical basis of the program also draws from other well‐established and empirically based psychotherapeutic approaches (eg, acceptance and commitment therapy (ACT), motivational interviewing). CAP provides educational content and introductory skills training that are similar to what would be obtained in the first 4‐6 individual sessions of cognitive behavioral therapy for pain treatment.

The key assets from a psychoeducational perspective are the parent and adolescent workbooks. In addition to being used during the workshop, the workbooks also provide home‐based parent‐adolescent activities focused on behavior change, enhanced functional ability, and further education and resources for coping and support, postworkshop. Concretely, some of the adolescent skills and tools draw from CBT (eg, thought restructuring, diaphragmatic breathing, guided imagery, goal setting); motivational interviewing (eg, exploring and building confidence); ACT (eg, identifying comfort, planning valued activities, mindfulness); biofeedback‐assisted relaxation (eg, use of heart rate rhythm monitoring); and social/peer support. For parents, the workbook outlines CBT‐based coping skills (eg, thought restructuring and cognitive flexibility, diaphragmatic breathing); ACT‐based identification of valued activities/experiences, mindfulness exercise and practice; parenting training (eg, communication skills training, reflective listening, behavior change principles and practice); and strategies for parenting a child with chronic pain (eg, activity pacing for return to function).

In addition to the adolescent and parent workbooks (approximately 65 pages each), CAP program assets also include clinical leader’s manual for each the adolescent and the parent programs (78 pages each). These manuals provide structured content for clinical administration, prompts for discussion within the group setting, and guidelines for managing potentially challenging clinical issues. They also provide suggested timelines for content delivery and resources for CAP clinicians.

### 2.2 Generating local implementation procedures

**Figure 3:**
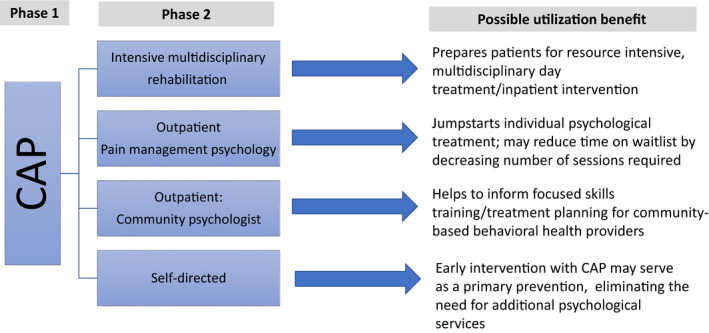
Comfort Ability Program (CAP) utilization

Figure 3 illustrates how engagement with CAP as a Phase 1 treatment could impact subsequent patient care (Phase 2) at various levels of the healthcare system. With this implementation roadmap, CAP was granted department‐level funding to test implementation of the intervention with 30 families. Early program assessment that incorporated parent, adolescent, and provider feedback led to modifications of clinical and administrative procedures, such as more active adolescent involvement (vs didactic practices) and enhanced recruitment resources via educating providers about how to introduce the program to patients and families. Fundamentally, this initial pilot testing confirmed that the program implementation procedure was feasible; adolescents of various ages (10‐17) and various types of pain (eg, abdominal, headache, neuropathic) along with their parents, could positively engage with a day‐long intervention that provided targeted knowledge and skills training. After testing the implementation procedures in this way, CAP moved into a wider‐scale evaluation phase.

## 3 EVALUATE CLINICAL AND SYSTEMS‐BASED PROCESSES AND REFINE CONTENT

By 2013, CAP was running at Boston Children’s Hospital eight times per year. As part of the program, adolescents and parents filled out preprogram baseline needs assessments and postprogram standardized and open response acceptability questionnaires. Positive patient, parent and provider feedback (Table 1) and improvements at the system level (eg, reduced pain psychology waitlists; enhanced engagement with patients in a wide catchment area) spurred additional program evaluation efforts. A small pain education grant that supported clinician time and research assistant support, facilitated a single‐arm feasibility study. Importantly, CAP had rapidly become an integrated clinical intervention within the Pain Treatment Service. As such, research needed to be conducted without disrupting the flow of patients who were accessing this service. Thus, the next phase of research assessed the CAP intervention in the context of the ongoing standard care delivered through the clinic.

**Table 1:** Focus on feedback – sample responses

Adolescent feedback
“One of the biggest things I gained from this experience was the chance to finally meet others who are going through something. It was wonderful because each of us had sort of felt … alone.” ~ age 13
“I got to learn different techniques that I could do by myself and that I could do with other people.” ~age 15

Parent feedback
“We can’t thank you all enough. We learned so much. Most importantly, my child sees that things will get better. The tools she gained in the workshop are helping her make daily steps to improving her pain.”
“This was so, so helpful! I’ve made so many notes today and learned so much. I’m going to go home and read my books again and I can’t wait to talk to my daughter about these things.”
“Thank you for everything; my child and I learned a lot. I am happy to report that my child has been functioning much better since The Comfort Ability!”
“My child continues his perfect school attendance streak since the program, despite headaches. We LOVE the scaffolded plans; they seem to be our lifeboat. THANK YOU.”
“This was an inspiring and useful experience for us and our daughter. As a matter of fact, after talking with the school nurse this morning, I was pleased to hear that my child had talked with her about techniques she learned [at the workshop].”

Provider feedback
“We cannot thank you enough for all of your support and time. This was a fabulous opportunity to refine our delivery and clinical skills, not only for CAP implementation moving forward, but for our ongoing work with patients and families who are affected by chronic pain.” ~ Yale Child Study Center
“Adopting the Comfort Ability program was a team effort. From our initial phone consultation with Dr. Coakley, through our on‐site training experience at Boston Children’s, further email and phone communications, to hosting our initial workshop and beyond, our Lurie Children’s team has benefited from the expertise of the Comfort Ability network. The Boston Children’s team provided resources and feedback for funding the program start‐up and maintenance and they offered a package of materials for program marketing, participant registration and tracking, and program evaluation. Bolstered by this strong external support, our team generated enthusiasm internally among colleagues and administrators.” ~ Lurie Children’s Hospital

### 3.1 Evaluate clinical and system‐based processes

Results from a single‐arm study of 120 families confirmed that CAP was a highly feasible and acceptable intervention.^16^ Additionally, adolescents and parents reported significant clinical gains. Within a week of completion of the workshop, both adolescent and parents reported greatly enhanced knowledge about how pain functions in the body and the role of psychology in pain management. Parents also reported reduced pain catastrophizing and over‐protectiveness in their parenting practices. Clinical gains were maintained even after three months of attending, with reported improvements in adolescent’s functional ability and adolescent pain catastrophizing.^16^ Subsequent research has also shown parental difference in perception of adolescent pain before and after attending CAP.^34^

At the systems level, to help sustain program growth, CAP instituted an out‐of‐pocket registration fee for participating families. The out‐of‐pocket fee per family was derived from estimating the 2013 average cost for family copay per therapeutic hour and then multiplying that by six to account for the six therapeutic hours in this intervention. This revenue offset approximately 70% of the program costs (ie, materials and clinician time) and had the additional benefit of reducing the no‐show rate from an average of 30% to an average of 15% after the implementation of the registration fee. The reduced no‐show rate was hypothesized to be a result of the family’s enhanced commitment by virtue of their payment for service. In other words, as a result of having to pay to attend the program, families seemed to commit to coming more often. To continue to maintain the goal of accessibility, department‐level support provided no‐fee scholarships to families who could not afford the registration fee.

### 3.2 Refine content and resources

The combination of the formal feasibility research and lived clinical experience running this program contributed to further modifications and expansion geared toward sustainability and growth. For example, within the parents’ workshop, hands‐on training and in vivo experience with relaxation strategies were included to augment the didactic aspects. In the adolescent program, the pain education portion was redesigned to include more visual aids, interactive learning procedures, and concrete examples, to address various learning styles and developmental needs. Additionally, strong patient interest for additional evidence‐informed resources and peer/social support promoted the creation of the CAP website.^35^ Funded by a healthcare innovation grant, the CAP website provides evidence‐informed resources (ie, embedded audio relaxation and mindfulness exercises; instructional “how to” videos to support school function, enhance parent‐child communication, and facilitate relaxation; downloadable psychoeducation materials); and social support via adolescent/parent stories and testimonials.

## 4 DEVELOP AND IMPLEMENT DISSEMINATION PROCEDURES WITH A SHARED FOCUS ON INSTITUTIONAL AND PROVIDER EXPERIENCE

**Figure 4:**
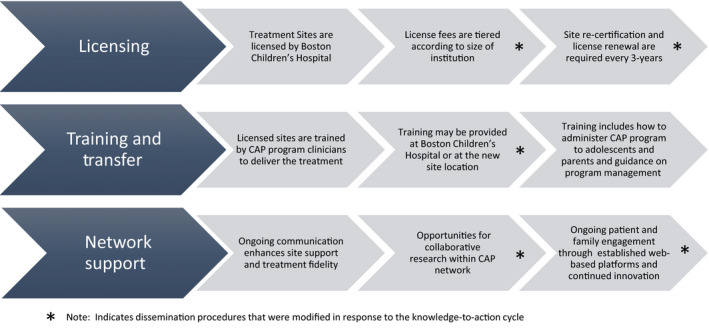
Overview of Comfort Ability Program (CAP) dissemination procedures

In 2014, CAP was approached by a Canadian children’s hospital requesting the resources and support to launch the program in their institution. At that time, based on the testing and modification cycle as described above, CAP had readiness to explore dissemination. In entering this phase of the knowledge‐to‐action dissemination cycle, CAP was focused on (a) generating a flexible protocol for knowledge transfer across various institutional and healthcare settings, and (b) ensuring the fidelity of CAP throughout the training and transfer procedures. Toward these shared objectives, administration manuals and procedures for training were created to anchor and standardize the intervention across settings and institutions. With this first partner site launch and based on the interest of three additional sites in the following year, formal CAP dissemination procedures were refined. Figure 4 outlines CAP’s established dissemination procedures, noting where procedures have been revised based on provider and institutional feedback. These modifications reflect the iterative knowledge‐to‐action cycle and are described below.

### 4.1 Institutional‐level considerations

Early in the transfer process, it became evident that institutional‐level commitment was needed to ensure successful program dissemination. Without this high‐level institutional support, the clinical demands, administrative procedures, and logistics associated with adopting a new program placed an unrealistic burden on new site directors that could jeopardize successful implementation and maintenance of CAP. This key realization was the impetus for CAP’s licensing procedures. The technology and innovation office at Boston Children’s Hospital thus generated a licensing agreement inclusive of usage fees and standardized practices for clinical training and transfer of materials. With this legal framework in place, stakeholders that had interest in partnering to implement CAP necessarily had to involve division or department heads as well as legal and finance departments. This system‐level involvement, while creating some additional effort at the onset of program adoption, was intended to pave the way for a streamlined knowledge transfer and to increase institutional accountability and commitment. When program transfer was supported by the department, division, and institution, all aspects of program transfer—including clinical training, acquisition of new resources, patient referrals, and program launch—were made more visible and had greater chance of full and successful adoption. Additionally, revenues from the licensing fees, in turn, could provide essential funding to support ongoing research and program development.

### 4.2 Provider‐level considerations

Concomitant with the standardization of CAP implementation and dissemination processes, additional focus was placed on the process of training clinicians in the CAP clinical content and procedures. CAP was written and designed by a pediatric psychologist with expertise in pain management. It was intended to amplify the clinical reach of pediatric psychologists embedded within healthcare systems by providing a highly structured, well‐designed group‐based program that honed and expanded the clinical skill set commonly used by pediatric psychologists. As such, CAP assumes an already high‐level of knowledge and clinical skill in pediatric psychology, though expertise in pain management, per se, is not required. Highly skilled pediatric psychologists can efficiently learn CAP content and procedures with a workshop observation and 4‐6 hours of direct training. When new sites are licensed, trained, and prepared to launch the program, a CAP‐licensed clinician provides live supervision of the first workshop and structured feedback to the team. A comprehensive report includes not only constructive clinical feedback, but also important feedback about the infrastructure and procedures that have been activated to support the program. Once a site has successfully trained CAP clinicians, they can generate future program leaders within their site by replicating the same training methodology.

Notably, consistent with the iterative knowledge‐to‐action cycle, the collaborative stakeholder relationships within and between CAP partner sites further informed dissemination procedures. For example, several sites requested additional supporting procedures and materials to further facilitate transfer. This prompted CAP’s creation of an “on‐boarding kit” inclusive of materials that clearly outline system‐level operations (ie, patient recruitment flyers and materials, checklists for materials management, patients’ acceptability rating scales, marketing materials, and press release). In this way, CAP reduced the individual stakeholders’ time and fiscal resources needed for successful adoption. These core CAP assets, in turn, became part of the standard dissemination protocols/materials.

In the context of CAP being a manualized intervention that requires clinician training and fidelity‐based licensing, the expectation is that the delivery of the intervention follows the guidelines provided in the group leader manual. However, partner sites are encouraged to share experiences and make suggestions to enhance the needs of a site’s patient population. The information gathered from partner sites is used for content revisions and program enhancements. While some of the suggestions are implemented quickly (ie, suggestion for a patient‐facing newsletter), other facets of partner site feedback are used to make more systematic changes, such as the clinical content revisions that are evident in the  soon to be produced second edition publication of the leader manual and adolescent/parent workbooks.

## 5 EXPAND CLINICAL, INSTITUTIONAL, AND PATIENT PARTNERSHIPS, AND BUILD ENHANCEMENTS

To date, CAP has created a network of 18 partnerships sites across the United States, Canada, and Australia.^36^ During this time of expansion (2015‐2019), CAP has continued to evolve in line with the iterative cycle of generation, dissemination, and sustainment of new knowledge^1^ based on the stakeholder needs. This phase of expansion and development touched every level of the knowledge‐to‐action cycle and included adjustments to program content, best practices for clinical delivery, and training and transfer procedures. To support this phase of expansion, in 2018, CAP hired a consultant with expertise in non‐profit  initiatives to create a development plan that augmented the skills of the pediatric psychologists who had researched, designed, and implemented all CAP‐related function to that point. This collaboration provided an essential framework to generate funding at the donor and institutional level and created the platform by which CAP could continue to expand and generate additional knowledge mobilization.

### 5.1 Clinical partnerships

The growing CAP network is now among CAP’s most valuable core assets. Building a network of shared knowledge users has generated essential opportunities for program enhancements and new ideas for innovation. CAP maintains connections with the partner sites with bidirectional communication between the CAP team and partner sites, as well as cross‐communication between partners. The growing network, however, also presented a tremendous system‐level challenge. In order to support the professional network, continue to build enhancements, and increase the number of patients served by the intervention at the local level, there was a need to expand the CAP clinical and development teams, a transition that required a comprehensive and strategic growth plan.

### 5.2 Institutional partnerships

The Comfort Ability Program has expanded across a variety of healthcare settings and cultures, both public (eg, Canada and Australia) and private healthcare systems, with a wide range of access to funding, clinical support, and patient needs. In each new setting, CAP seeks to engage in collaborative problem solving to determine what contexts may help to optimally support implementation and maintenance. Funding mechanisms range from institutional‐level start‐up grants, affiliation with university or community centers, institutional funding, various levels of patient‐pay obligations, donor support, or a combination of the above.

Given the expressed interest of current CAP network to administer the workshop to Spanish and French‐speaking communities, CAP is currently exploring the translation of the manuals and the development of a dissemination model of CAP to non‐English‐speaking populations. CAP is also diversifying through its inclusion of youth with disabilities. In 2020, CAP published a braille translation of the workbooks to accommodate patients with visual impairment. Notably, full inclusion of children or parents with visual impairment requires administrative, clinical, and cultural modifications of the program; developing, testing, and then sharing these resources with partner sites as CAP has done, further reflect the knowledge‐to‐action cycle. In a similar way, CAP hopes to establish international collaborations with pediatric psychologists and pediatric institutions from other continents (eg, Europe and Asia), recognizing that in some cases these initiatives will require a new knowledge‐to‐action cycle starting with the identification of unique gaps in care, analyses of barriers, and exploration of cultural differences.

Recognizing that the burden of sustaining any clinical innovation is the demonstration of fiscal viability and that demonstrated cost‐effectiveness can enhance opportunities for continued funding for existing sites and create “buy‐in” for new sites, CAP sought and was awarded state‐funding through the Delivery Systems Reform Incentive Program.^37^ This grant is focused on the systematic evaluation of health costs associated with program operations and on the healthcare utilization of patients who completed the workshop as compared to patients who were referred to but did not participate in the program due to access barriers (eg, transportation limitations and work demands, etc.). The goal of this ongoing research is to evaluate the CAP cost‐utility to institutions, individual families, and public insurance plans alike via evaluations of program cost per person as it relates to healthcare utilization, direct healthcare expenses, ancillary healthcare expenses, and health‐related quality of life pre‐ and postintervention. Demonstration of cost‐utility in one or more domains is essential to enhance wide‐scale adoption of CAP in addition to furthering the positive impact of the workshop at the patient and family level.

### 5.3 Patient partnerships

Given the growing network of patients and families benefiting from CAP, coupled with the ongoing valuable input from workshop participants to CAP development, another key program enhancement was launched in 2017: the formal patient and parent advisory boards. Board members are graduates of CAP and participate on a volunteer basis. They work collaboratively to suggest and produce new content, generate resources, and advise the CAP clinical and research programming. Members from the CAP advisory boards also provide direct social support to patients through monthly online peer/parent mentorship chats and often serve as guest speakers, either in person or through a virtual platform, for network partners during CAP workshops. Importantly, adolescents and parents serve limited appointments on the advisory boards, set their own agendas, and are responsible for vetting new member candidates. In this way, CAP seeks to promote an autonomous board that offers new, patient‐ and family‐lived perspectives and ideas for addressing the knowledge gaps and creating new resources for the psychological treatment of persistent pain. The patient and parent advisory boards are now another of the core CAP assets.

Additional CAP enhancements have focused on increased accessibility to patient‐centered postworkshop education and support. For example, CAP generates a monthly e‐newsletter, has interactive features on the website (eg, “ask the expert” question submissions, options to share patient stories, and psychologist moderated online chats). Moreover, CAP began active engagement with social media in 2017 (ie, Twitter, Facebook, and Instagram) to share evidence‐informed content relevant to CAP’s mission. To further knowledge mobilization, CAP has been actively involved in media and public relations. Articles in popular press (eg, Washington Post,^38^ New York Times,^39^ The Atlantic^40^) that feature CAP have been instrumental in terms of educating adolescents with chronic pain and their parents about available treatments and supports, increasing patient referrals at the national level and spearheading new patient and clinical partnerships.

### 5.4 Build enhancements

As a way to innovate, generate, and disseminate new knowledge, CAP is actively working on several projects, such as developing and testing disease/entity specific modules to enhance applicability (eg, youth with sickle cell disease, oncological processes,  or gastrointestinal illness); expansion of online communities and peer mentorship programming; extending and diversifying CAP’s peer and parent advisory boards; and increased visibility via ongoing education of professionals working with children who have chronic pain not only in the medical and mental health fields, but also in the community (eg, teachers). Moreover, in response to the COVID‐19 pandemic, CAP has mobilized to generate additional online resources such as webinars for parents and teens with pain. Additionally, CAP is piloting a virtual telehealth modification of the program this year.

## 6 MONITOR AND SUSTAIN KNOWLEDGE USE, BUILD NETWORK RELATIONSHIP, AND CONTINUED INNOVATION

With a commitment to maintain and grow knowledge partners, CAP is simultaneously focused on sustaining knowledge use, creating opportunities to expand its network, and creating new innovations that foster improved clinical care in the psychological management of chronic pediatric pain. In addition to the evidence‐based content and the patient/family‐centered approach, CAP’s systematic administration plan has made it an attractive clinical service for many institutions.

### 6.1 Monitor and sustain knowledge use

Given that evidence‐based knowledge and best practices in the field continue to evolve, sustained knowledge use as it pertains to CAP is not a static process. CAP requests that all sites maintain treatment fidelity with intermittent self‐assessment using a CAP fidelity checklist. More substantially, as CAP license agreements were initially five years in duration, many of CAP’s early partner sites will soon be ready for recertification. During this process, CAP will more fully assess clinical and implementation procedures and determine if more structured monitoring may be needed to identify areas of program drift and ensure  that new materials are fully integrated.

Importantly, CAP can only maintain established institutional commitments and a strong network of trained psychologists by actively evolving the program. For example, in the five years since the program was first disseminated, best practices for neuroscience education, a key part of the program for adolescents and parents, has shifted.^41^ With a stronger evidence base and consensus on key elements of education that are known to contribute to increased function,^42^ CAP is currently revising the pain education module within the program. When this module is complete and reflects the current state of the art in this area, it will be reviewed by the  peer and parent  advisory boards, pilot tested at the local level, shared for comment across the network of CAP providers, and finally integrated into the program. New materials will replace existing content and will be shared with existing sites, and virtual training will be offered to promote implementation. In this way, CAP can produce a knowledge‐to‐action network and a knowledge dissemination highway reducing the individual clinical and research burden of each psychologist embedded within each institution. Furthermore, CAP remains a highly valued healthcare commodity, extending  scarce resources while continuing to ensure that evidence‐based intervention is widely accessible.

### 6.2 Build network relationships

The creation of a solid professional network with available consultation opportunities is essential for collaborative research, content development, and additional innovation. Recently, a Canadian national level collaboration has been initiated between CAP and Solutions for Kids in Pain (SKIP),^43^ a knowledge mobilization network. Given the successful track record of CAP for cross‐institutional implementation and sustainability, CAP is well‐poised to join forces with SKIP in their effort to bridge the gap between current treatment practices and available evidence‐based solutions for children with pain in Canada. At the international‐level, CAP has an established partnership with Childkind,^44^ an organization whose mission is to help medical institutions across the world identify gaps in pain medicine practice and implement evidence‐based solutions. These key partnerships can greatly extend and enhance CAP’s clinical reach, promoting knowledge mobilization at a macro level.

### 6.3 Continued innovation

In 2019, the CAP team again expanded, preparing for the next phase of growth and innovation. Relying on CAP’s well‐established knowledge to action cycle, CAP began 2020 with a formal needs ‐ based assessment across the network of partner sites (eg, What is currently going well? What barriers impede CAP implementation? What practice‐informed solutions do you propose?, etc.) and system‐level research pertaining to emerging projects (eg, How do psychologists’ licensing laws differentially impact patient‐facing virtual technology across states and continents?). This information will be shared back with the CAP stakeholders and used broadly to grow and enhance the full array of CAP assets.

Additionally, as described earlier in the article, CAP is in the process of evaluating in more detail in what ways the program may provide clinical benefit to an institution by reducing barriers to care, enhancing patient’s recovery, and facilitating cost‐effective healthcare utilization. This research initiative, supported by a Delivery System Reform Incentive Payment Program (DSRIP) Innovation Grant,^37^ is awarded to providers in the USA to undertake research that can lead to healthcare delivery transformation efforts. The specific research foci are (a) to evaluate healthcare utilization and direct and ancillary costs that are associated with healthcare utilization before and after attending the CAP workshop; and (b) to identify risk factors that characterize the healthcare utilization patterns in adolescents with chronic pain that attend the CAP workshop. These lines of implementation research will help to inform further programmatic developments and will increase the likelihood that all stakeholders can maximally benefit from CAP’s evolving innovations and resources.

## 7 CONCLUSION

The Comfort Ability Program was developed in 2011 from the ground up to mobilize the psychology research evidence base for pediatric chronic pain, moving essential skills and strategies into accessible clinical practice. The goal was to maximize impact by aligning the clinical intervention with the strongest evidence in the field, carefully attending to patient‐identified access barriers, and developing implementation procedures that could be systematically replicated. By adhering to an implementation science paradigm, such as the knowledge‐to‐action cycle, CAP has evolved into an intervention platform with a wide reach and growing capacity.

In addition to its evidence‐based content and patient‐informed approach, the program’s systematic administration plan makes it an attractive clinical service for many institutions. CAP has both a well‐established licensed model for intervention and a standardized model for cross‐institution training and knowledge transfer. The training model includes content and instruction for administering CAP to adolescents and parents, supervision and direct feedback, as well as ongoing guidance and consultation on program management. The CAP professional network and consultation team provides ongoing communication with site clinical directors, enhances site support, and promotes treatment fidelity. Additionally, CAP provides opportunities for collaborative research, content development, and intervention, thus further enhancing knowledge mobilization.

The rapid expansion of CAP as a knowledge dissemination tool illustrates the value of this clinical research paradigm and offers a viable roadmap toward mobilizing knowledge in other areas of clinical care. While CAP has demonstrated success thus far, ongoing innovation, clinical and research synthesis, delivery refinement, and program evaluation are continually needed to inform the iterative knowledge‐to‐action cycle.

## CONFLICT OF INTERESTS

There is no conflict of interest. CAP is a non‐profit program licensed through Boston Children’s Hospital. A portion of the licensing fees support the research laboratory and staff salary.

## AUTHOR CONTRIBUTIONS

R.C. developed the idea, designed the theoretical framework, and drafted the manuscript. S.B contributed to the writing, provided critical feedback, and helped to shape the final version of the submission.
